# Longitudinal Displacement for Left Ventricular Function Assessment

**DOI:** 10.3390/jcdd12020053

**Published:** 2025-01-31

**Authors:** Marina Leitman, Vladimir Tyomkin

**Affiliations:** 1 Department of Cardiology, Shamir Medical Center, Zerifin 70300, Israel; 2 Sackler School of Medicine, Tel Aviv University, Tel Aviv 6997801, Israel

**Keywords:** left ventricular function, longitudinal displacement, speckle tracking imaging, longitudinal strain

## Abstract

Background: Quantitative evaluation of myocardial function traditionally relies on parameters such as ejection fraction and strain. Strain, reflecting the relative change in the length of a myocardial segment over the cardiac cycle, has been extensively studied in various cardiac pathologies over the past two decades. However, the absolute length change, or longitudinal displacement, of myocardial segments during the cardiac cycle has received limited attention. This study aims to evaluate longitudinal displacement in two separate groups: healthy athletes and patients with left ventricular dysfunction, providing new insights into myocardial function assessment. Methods: Echocardiographic examinations were performed on 30 healthy football players and 30 patients with left ventricular dysfunction using speckle-tracking imaging analysis. Global and regional peak longitudinal displacement values were calculated and compared with corresponding global and regional peak longitudinal strain measurements. A manual alternative for calculating global longitudinal strain was also proposed. Results: An inverse correlation was found between regional longitudinal displacement and regional longitudinal strain. Longitudinal displacement was maximal in the basal segments and lowest in the apex of the left ventricle, exhibiting a reversed basal-to-apical gradient (17.6 ± 3.5 mm vs. 11.5 ± 2.9 mm vs. 4.22 ± 1.7 mm in basal, mid, and apical segments, respectively; *p* < 0.000001). Maximal longitudinal displacement was observed in the inferior and posterior walls of the left ventricle. In the 30 patients with left ventricular dysfunction, global longitudinal displacement was significantly lower than in healthy individuals (4.4 ± 1.7 mm vs. 11.7 ± 1.5 mm, *p* < 0.000001). Global longitudinal displacement and global longitudinal strain showed a strong negative correlation (r = −0.72, *p* < 0.000001). Manually calculated global longitudinal strain demonstrated good agreement with speckle-tracking-based global longitudinal strain. Conclusions: Peak longitudinal displacement can be used to evaluate both regional and global myocardial function, similarly to peak longitudinal strain. Unlike strain, longitudinal displacement exhibits a reversed basal-to-apical gradient, with the highest values at the base of the left ventricle and the lowest at the apex. Global and regional longitudinal displacement is significantly reduced in patients with left ventricular dysfunction. Global longitudinal strain can be manually calculated using displacement measurements. Further studies are needed to evaluate peak longitudinal displacement in various cardiac pathologies.

## 1. Introduction

Assessment of left ventricular function has traditionally been carried out through the evaluation of ejection fraction. Visual estimation of ejection fraction is accomplished by the calculation of ejection fraction, which can be performed manually using Simpson’s formula or semi-automatically, including the 3D method, or 2D automatic ejection fraction calculation using speckle-tracking imaging [1]. The same speckle-tracking imaging software allows for the calculation of global longitudinal strain, which is often reported when precise measurements of left ventricular function are required. Ejection fraction primarily holds prognostic value in cases of moderate to severe left ventricular dysfunction, particularly when it falls below 40% [2]. In cases of mild left ventricular dysfunction or when ejection fraction is preserved, global longitudinal strain provides additional incremental value, particularly when EF is above 35%. However, there is no exact direct or linear correlation between ejection fraction and longitudinal strain, with overlap observed between different groups [2]. In patients undergoing chemotherapy, global longitudinal strain is essential for the early diagnosis and prevention of chemotherapy-induced cardiomyopathy [3,4,5]. In patients with cardiac amyloidosis, longitudinal strain can detect early myocardial changes [6]. Strain represents relative systolic myocardial shortening, whether regional or global. In contrast to global longitudinal strain, regional myocardial strain is not widely utilized. Strain is a calculated parameter and is not measured directly. To gain a deeper understanding of the physiology underlying strain and to identify the parameters complementary to the established methods of left ventricular function assessment, this study measured the mechanical and physiological “precursor” to strain, known as longitudinal displacement. This parameter was then compared with strain to assess its potential utility in myocardial function evaluation in two separate groups: healthy athletes and patients with left ventricular dysfunction.

## 2. Materials and Methods

### 2.1. Study Population

A total of 30 healthy football players [Group 1] underwent routine check-ups at our hospital in 2024. Echocardiographic examinations were retrieved and analyzed offline, with global and regional longitudinal left ventricular strain calculated from apical 4-chamber, 2-chamber, and 3-chamber views. A visual plot of the three-plane strain was generated for each patient and analyzed in detail to assess regional strain differences. Longitudinal displacement, both global and regional, was similarly obtained from the same views. A visual plot of three-plane longitudinal displacement was generated and analyzed. Global and regional longitudinal displacement were compared with global and regional longitudinal strain.

Additionally, echocardiographic examinations of 30 patients with left ventricular dysfunction [Group 2] were analyzed using a similar speckle-tracking imaging approach, with calculations of both longitudinal displacement and strain.

### 2.2. Echocardiographic Assessment

All echocardiographic exams were performed using the Vivid E95 (General Electric; Horten, Norway) with a standard transducer frequency of 1.7–4 MHz. The frame rate during echocardiographic examinations was at least 40 frames per second. Comprehensive transthoracic echocardiographic examinations were performed in accordance with the latest recommendations for chamber quantification [1]. In brief, linear, volumetric, and Doppler measurements were performed. Standard echocardiographic views were acquired, including the parasternal long and short axis at three levels—basal, mid-ventricular, and apical—as well as apical 4-chamber, 2-chamber, and 3-chamber views. Diastolic function was assessed in accordance with the current recommendations [7]: E wave amplitude, A wave amplitude, E/A ratio, E wave deceleration time, tissue Doppler E’ septal velocity [E’s], and tissue Doppler E’ lateral velocity [E’l].

The biplane left atrial volume index (LAVi) was calculated using the following formula:

$$\frac{8}{3}\pi \times \frac{\left(A1\right)\times \left(A2\right)}{L}$$

where A1 and A2 represent the areas of the left atrium obtained from the apical 4-chamber and 2-chamber views, respectively, and L is the shortest vertical dimension of the left atrium. LAVi was normalized to body surface area. The left ventricular mass index (LVMi) was calculated using the following formula:

$$0.8\times 1.4\times \left[{\left(IVS+PW+LVID\right)}^{3}-{LVID}^{3}\right]+0.6\;g$$

where IVS is the interventricular septal thickness at end-diastole, PW is the posterior wall thickness at end-diastole, and LVID is the left ventricular internal diameter at end-diastole. LVMi was normalized to body surface area.

All echocardiographic examinations were then transferred to the EchoPAC workstation (Version 204) for further offline speckle-tracking imaging analysis. Speckle-tracking imaging analysis was performed according to the original recommendations using the apical views. [8]. Aortic valve closure on the apical long-axis view served as a reference for end-systole, which was verified by the aortic Doppler flow recorded from the apical five-chamber view [8]. Offline speckle-tracking analysis was performed to calculate global and regional longitudinal displacement, as well as global and regional longitudinal strain.

#### 2.2.1. Specification Regarding Longitudinal Deformation Parameters: Strain and Displacement 

**Figure 1:**
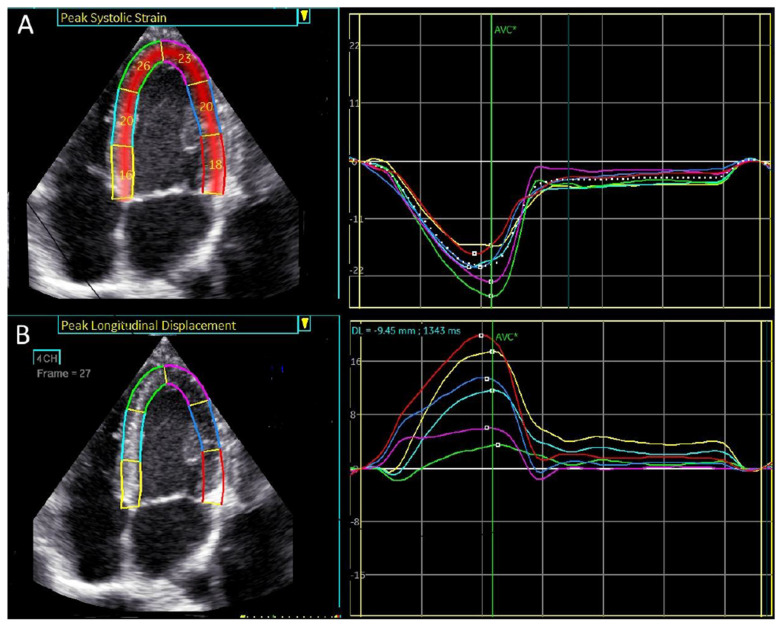
4-chamber apical view. Regional longitudinal strain and displacement of a normal subject. (**A**) Regional peak systolic longitudinal strain has a negative direction. (**B**) Regional peak systolic longitudinal displacement has a positive direction.

Strain in Figure 1 is a relative deformation, defined as the ratio of the change in length to the original length, and is expressed as a percentage [8].

Strain is calculated using the following formula:

Strain = (L(t) − L(0))/L(0)

where L(0) is the initial length and L(t) is the new length at a specific time. Longitudinal strain is typically negative because the left ventricle shortens during systole (Figure 1A).

Longitudinal displacement is the absolute change in length, is measured in millimeters (mm), and is calculated using the following formula:

Displacement = L(t) − L(0)

where L(0) is the initial length and L(t) is the new length.

Typically, longitudinal displacement in the left ventricle has a positive direction (Figure 1B).

#### 2.2.2. Method for Manual Global Longitudinal Strain Measurement (GLSm)

Based on the definitions of strain and displacement, we conducted manual measurements of the distances between the mitral annulus plane and the apex of the left ventricle at diastole and systole across all three apical views (4-chamber, 2-chamber, and 3-chamber).

**Figure 2:**
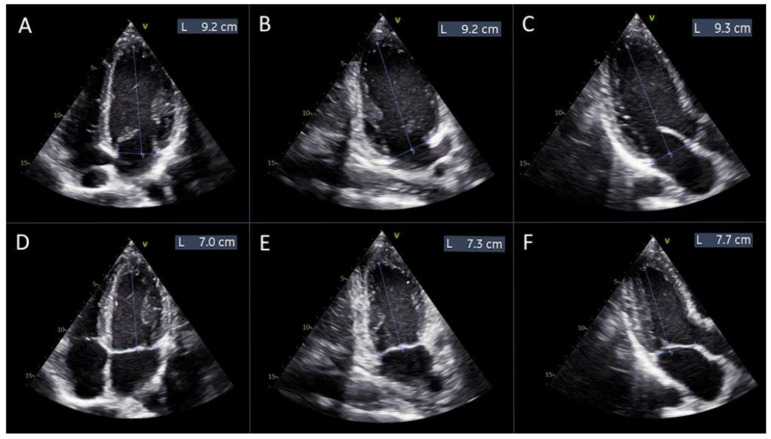
Measurements of left ventricular length for manual global longitudinal strain calculation. Panels (**A**–**C**) display the measurements of the left ventricular length obtained from the apical 4-chamber, 2-chamber, and 3-chamber views, respectively, at diastole. Panels (**D**–**F**) show the corresponding measurements from the same apical views at systole.

Global left ventricular displacement was calculated as the difference in left ventricular length between diastole and systole, averaged across the three apical views. Subsequently, the average longitudinal displacement was divided by the average diastolic left ventricular length to derive the manual global longitudinal strain (GLSm), calculated using the following formula:

GLSm = (((L1d + L2d + L3d)/3) − ((L1s + L2s + L3s)/3))/((L1d + L2d + L3d)/3) × 100

where L1d, L2d, and L3d are the distances from the mitral annulus plane to the apex of the left ventricle in diastole, measured in the 4-chamber, 2-chamber, and 3-chamber views, respectively (Figure 2A–C).

L1s, L2s, and L3s are the distances from the mitral annulus plane to the apex of the left ventricle in systole, measured in the same views (Figure 2D–F).

The manually calculated strain was compared with another recently described method for manual global longitudinal strain measurements, as outlined in reference [9].

### 2.3. Statistical Methods

Descriptive statistics were computed to summarize the characteristics of each parameter. The continuous data are presented as means ± standard deviations. The normal distribution of differences was assessed using the Kolmogorov–Smirnov test. A two-tailed dependent *t*-test was applied to continuous variables, while categorical data were reported as numbers and percentages. Univariate analysis was conducted using the Chi-Square test or Fisher’s exact test, as appropriate, to identify significant variables (*p* < 0.05). Statistical analysis was performed using IBM SPSS Statistics for Windows, Version 28.0 (IBM Corp, Armonk, NY, USA).

### 2.4. Ethical Approval

Formal approval from the Ethics (Helsinki) Committee at Shamir (Assaf Harofeh) Medical Center was not required for this retrospective study. All methods were conducted in accordance with relevant guidelines and regulations.

## 3. Results

The demographic and conventional echocardiographic data of the normal healthy subjects, Group 1, are presented in Table 1. The mean age was 24.6 years, and all standard echocardiographic parameters were within the normal limits. A strong negative correlation was found between peak systolic global longitudinal displacement and peak systolic global longitudinal strain, with a correlation coefficient of −0.78 (*p* < 0.000001).

**Figure 3:**
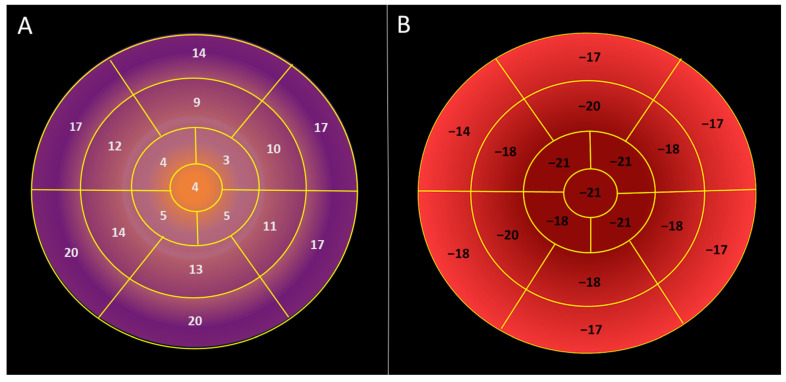
Visual plot of regional longitudinal displacement and strain over the left ventricle across the healthy study population [Group 1]. (**A**) Regional peak systolic longitudinal displacement over the left ventricle is highest in the basal segments and lowest in the apical segments. (**B**) Regional peak systolic longitudinal strain is lowest in the basal segments and highest in the apical segments.

Regional longitudinal displacement was highest in the basal segments and lowest in the apical segments, 17.6 ± 3.5 vs. 11.5 ± 2.9 vs. 4.22 ± 1.7 mm in basal, mid, and apical segments, respectively, *p* < 0.000001 (Table 2, Figure 2). In contrast, the lowest longitudinal strain was observed in the basal left ventricular segments, while the highest was found in the apex of the left ventricle, −16.3 ± 4.7 vs. −19 ± 2.5 vs. −21.1 ± 4.6% in basal, mid, and apical segments, respectively, *p* < 0.000001, (Table 2, Figure 3).

**Figure 4:**
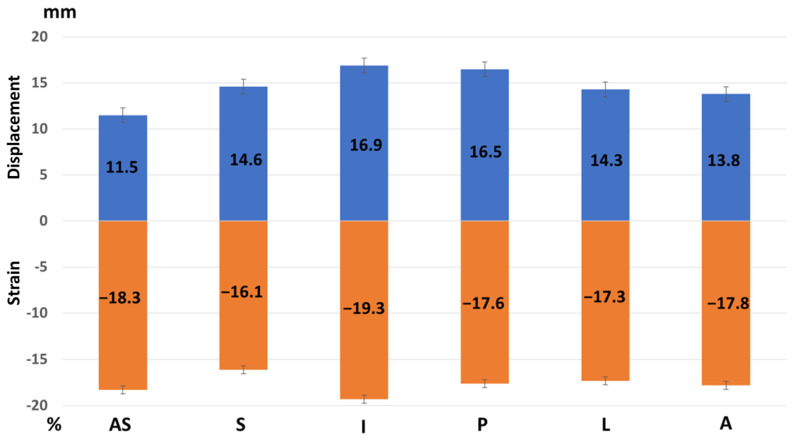
Regional peak systolic strain and longitudinal displacement of the left ventricle in a healthy study population [Group 1]. AS—antero-septal wall, S—septum, I—inferior wall, P—posterior wall, L—lateral wall, and A—anterior wall. Longitudinal displacement is highest in the inferior and posterior wall and lowest in the anterior septum. Longitudinal strain is highest in the inferior wall.

Peak longitudinal displacement was highest in the inferior and posterior segments, while it was lowest in the antero-septal wall (Table 3, Figure 4).

Peak longitudinal strain was highest in the inferior wall (Table 3, Figure 4).

**Figure 5:**
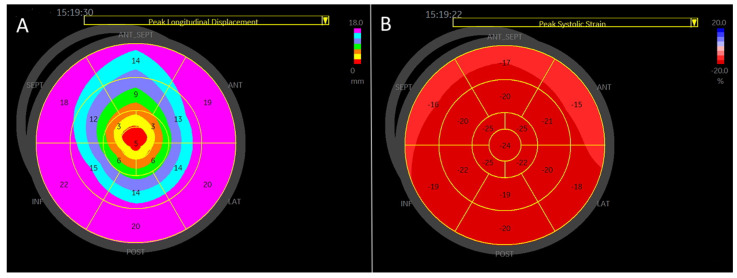
A visual plot of the normal peak longitudinal displacement and strain of 27-year-old football player. (**A**) Peak longitudinal displacement is highest in the basal segments and lowest in the apex. (**B**) Peak longitudinal strain is lowest in the basal segment and highest in the apex.

An example of peak systolic regional longitudinal displacement and strain for a 27-year-old football player is shown in Figure 5A,B. In contrast to the peak systolic strain, which exhibits a basal-to-apical gradient, peak longitudinal displacement is highest in the basal segments and lowest in the apical segments, demonstrating a reversed basal-to-apical gradient.

**Figure 6:**
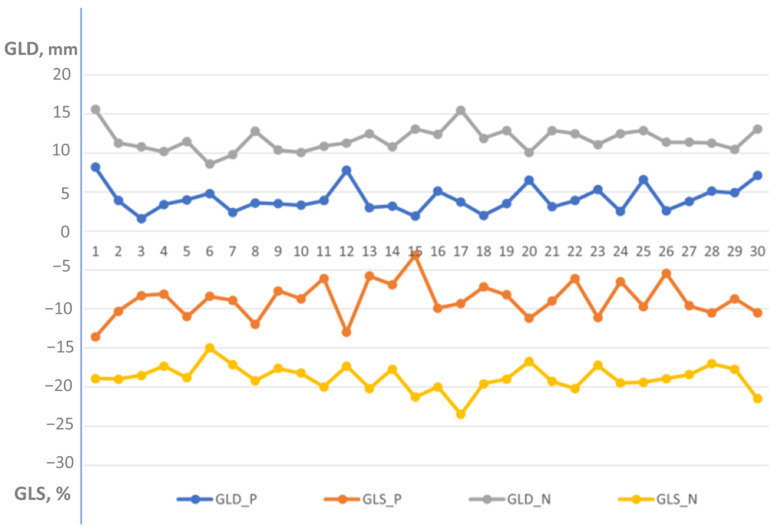
Distribution of global longitudinal displacement and global longitudinal strain in patients with left ventricular dysfunction [Group 2] versus healthy individuals [Group 1]. GLD-P, blue—global longitudinal displacement in patient group; GLS-P, orange—global longitudinal strain in patient group; GLD-N, gray—global longitudinal displacement in normal group; and GLS-N, yellow—global longitudinal strain in normal group. Global longitudinal displacement and strain were significantly lower in the patient group compared to the normal healthy individuals (4.4 ± 1.7 mm vs. 11.7 ± 1.5 mm, *p* < 0.000001 and −8.2 ± 2.3% vs. −18.8 ± 1.6%, *p* < 0.000001, respectively).

Echocardiographic examinations of patients with left ventricular dysfunction [Group 2] were analyzed separately in Table 4. The mean age was 69.1 ± 14.1 years, with 23 males (77%). The mean ejection fraction was 33.9 ± 3.2%. These patients had ischemic heart disease, with 19 having acute myocardial infarction and 11 having a history of old myocardial infarction. Global longitudinal displacement and strain were significantly lower in the patient group [Group 2] compared to the normal group [Group 1] (4.4 ± 1.7 mm vs. 11.7 ± 1.5 mm, *p* < 0.000001 and −8.2 ± 2.3% vs. −18.8 ± 1.6%, *p* < 0.000001, respectively) (Figure 6).

A strong negative correlation was found between global longitudinal displacement and global longitudinal strain (r = −0.72, *p* < 0.00001) in Group 2.

A detailed segmental longitudinal displacement analysis was performed for the healthy subjects and for patients with left ventricular dysfunction [Table 5]. The fifth percentile displacement values serve as the threshold for defining abnormal displacement. 

Additionally, segmental strain analysis was performed for both the healthy group and the group of patients with left ventricular dysfunction [Table 6]. 

Segmental displacement and strain are both reduced in patients with left ventricular dysfunction.

**Figure 7:**
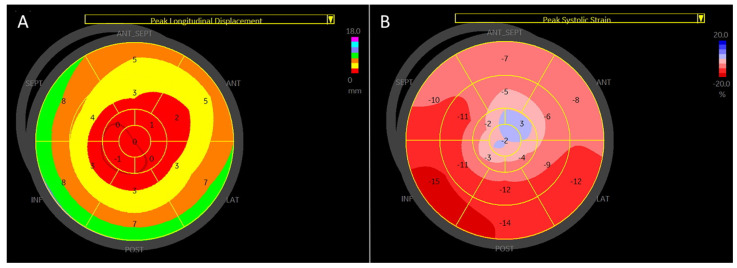
A visual plot of peak longitudinal displacement and strain in a 48-year-old man with anterior myocardial infarction and an ejection fraction of 35%. (**A**) Peak longitudinal displacement is very low (around 0) in the apical region (red), consistent with extensive apical myocardial infarction and involvement of other myocardial walls. Higher displacement is observed in the infero-posterolateral segments (green and brown). The average global longitudinal displacement is 3.4 mm. (**B**) Peak longitudinal strain is also very low in the corresponding apical segments, with better strain observed in the postero-lateral wall. The global longitudinal strain is −8.1%.

An example of a visual plot of longitudinal displacement and strain in a 48-year-old patient with moderate left ventricular dysfunction is shown in Figure 7.

**Figure 8:**
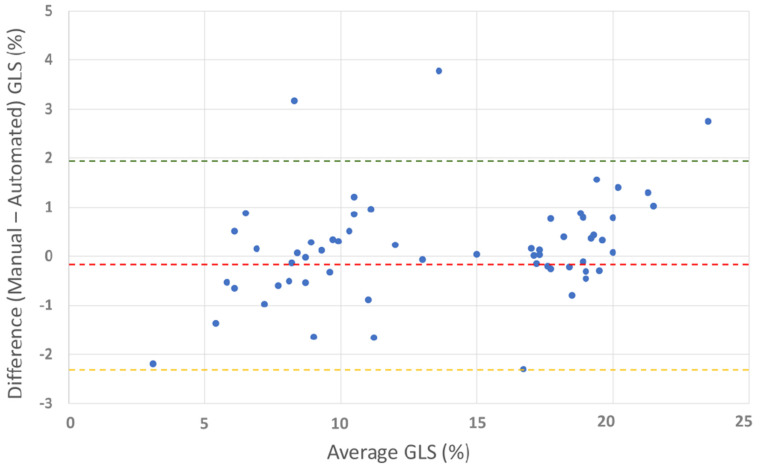
Bland–Altman plot for manual vs. automated global longitudinal strain. The plot shows the agreement between manual and automated GLS measurements. Red line: mean difference (−0.18%), green line: upper limit of agreement (1.92%), and orange line: lower limit of agreement (−2.29%). The Bland–Altman plot illustrates good overall agreement between manual and automated GLS methods, with a small mean difference (−0.18%). Most differences fall within the limits of agreement, confirming acceptable variability.

Manual measurements of global longitudinal strain were performed in both groups. Manually calculated global longitudinal strain demonstrated strong agreement with speckle-tracking-based global longitudinal strain [Figure 8], with a small mean difference (−0.18%).

We compared the manually calculated global longitudinal strain (GLSm) with the previously validated manual method [9] (GLSm(r)) and the GLS obtained using EchoPAC software [Table 7].

All three methods show highly consistent GLS values for healthy subjects, with small variations: GLSm (−18.5 ± 1.8%), GLSm(r) (−18.3 ± 1.8%), and GLS (−18.8 ± 1.6%). In patients with left ventricular dysfunction, the GLS values are significantly reduced, as expected, across all methods: GLSm (−8.9 ± 2.1%), GLSm(r) (−8.9 ± 2.0%), and GLS measured using the EchoPAC software (−8.8 ± 2.0%). The lower limit of normal for GLS is similar across all methods: −14.9% (GLSm), −14.7% (GLSm(r)), and −15.6% (GLS measured using the EchoPAC software). This uniformity suggests that the proposed method is reliable for detecting deviations from normal GLS values. All methods identify 100% of LV dysfunction patients as having abnormal GLS values when using a threshold of strain < −16%.

## 4. Discussion

In this study, we evaluated regional left ventricular longitudinal displacement in two groups: healthy football players, Group 1, and patients with left ventricular dysfunction, Group 2. We found that normally, longitudinal displacement is highest in the basal segments of the left ventricle and lowest in the apical segments, in contrast to longitudinal strain. Longitudinal displacement has not been extensively evaluated. Lind et al. [10] investigated the impact of isovolumic longitudinal displacement in healthy subjects using tissue Doppler imaging. Controversial results have been reported when measuring left ventricular longitudinal displacement using tissue Doppler imaging and M-mode in healthy subjects [11]. Assessment of longitudinal left ventricular shortening using tissue Doppler imaging has typically focused on measuring myocardial velocities in the basal and mid-ventricular segments [12]. In patients with heart failure and reduced systolic function, global and inferior longitudinal displacement were found to be independent predictors of all-cause mortality. Furthermore, inferior longitudinal displacement has been shown to be a significant prognostic indicator when compared with all conventional echocardiographic parameters. Consistent with previous studies, longitudinal displacement in this study was measured in the basal segments over the mitral annulus [13].

A simplified measurement of longitudinal shortening, assessed by M-mode echocardiography as mitral annular plane systolic excursion (MAPSE), has been shown to correlate with global longitudinal strain [14]. This parameter has also been confirmed by cardiovascular magnetic resonance [15,16] and 3D echocardiography [17]. With the introduction of speckle-tracking imaging, longitudinal strain has replaced tissue Doppler imaging in the evaluation of systolic myocardial function. Longitudinal strain and displacement are integral components of the complex myocardial mechanics involved during cardiac contraction [18]. While global and regional longitudinal strain have been extensively investigated in echocardiography and are now included in leading recommendations [1,2,3,4,5,6,8,19,20], longitudinal displacement across the left ventricle, both regional and global, have not yet been reported in echocardiography. In our study, we investigated longitudinal displacement across the left ventricle in a healthy population of football players, noting that global longitudinal strain is not affected by exercise in healthy subjects [21]. Longitudinal strain exhibits a basal-to-apical gradient, with the highest values at the apex of the left ventricle and the lowest at the base [22]. Unexpectedly, longitudinal displacement over the left ventricle demonstrated reversed basal to apical gradient, which was highest in the base and lowest in the apex. This indicates that relative displacement, which is strain, and absolute displacement are not the same. This suggests that complex forces contribute to the strong apical contraction, even though the apex has minimal longitudinal motion, is almost immobile, and represents the most stable anatomical region in the left ventricle. Longitudinal displacement is greatest in the basal segments, indicating that, of all cardiac segments, the basal segments travel the longest distance toward the apex during the cardiac cycle. We also found the highest longitudinal displacement in the inferior and posterior segments. The infero-basal segment, which may appear to be hypokinetic during echocardiographic examinations [23], can lead to false positive findings. Longitudinal displacement is a direct measurement and may be less prone to artifacts compared to other direct echocardiographic measurements. The calculated visual plot of longitudinal displacement shows well-ordered data (Figure 2 and Figure 4), in contrast to the visual plot of myocardial strain, which represents calculated data rather than a direct measurement.

In this study, we found that global longitudinal displacement like longitudinal strain is reduced significantly in patients with left ventricular dysfunction, adding new insights into the pathophysiology of myocardial contraction. A visual plot of longitudinal displacement (Figure 6) can provide additional insight into myocardial function assessment.

The manual calculation of global longitudinal strain (GLS) proposed in this study is a feasible and accurate alternative that is particularly valuable in clinical settings, where advanced speckle-tracking imaging software is either unavailable or inapplicable. This approach provides a reliable method for assessing cardiac function, enabling clinicians to evaluate GLS effectively with minimal dependence on specialized tools.

## 5. Limitations

Our study included 30 healthy individuals with normal left ventricular function and 30 patients with left ventricular dysfunction due to myocardial infarction. Further studies involving other cardiac pathologies are warranted to broaden the understanding of longitudinal displacement in different conditions.

## 6. Conclusions

Global and regional longitudinal displacement can serve as additional tools for evaluating myocardial function. Longitudinal displacement is typically highest in the basal segments and lowest in the apical segments of the left ventricle, with normal displacement being particularly pronounced in the inferior and basal segments. In patients with left ventricular dysfunction, longitudinal displacement is significantly reduced and can be used as an additional tool for myocardial function assessment. Global longitudinal strain can be manually calculated using displacement measurements. Further studies involving larger populations and various cardiac pathologies are encouraged.

## Figures and Tables

**Table 1:** Demographic and echocardiography characteristics of healthy athletes [Group 1]. BSA—body surface area, LAVi—left atrial volume index, LVEDD—left ventricular end diastolic diameter, LVESD—left ventricular end systolic diameter, IVS—interventricular septum, PW—posterior wall, LVMi—left ventricular mass index, EF—ejection fraction, GLS—global longitudinal strain, and GLD—global longitudinal displacement.

Age, years	24.6 ± 4.6
Height, cm	180.4 ± 8.3
Weight, kg	75 ± 6.3
BSA, m^2^	1.9 ± 0.1
LAVi, mL/m^2^	33.9 ± 8.7
LVEDD, cm	5.0 ± 0.4
LVESD, cm	3.1 ± 0.4
IVS, cm	1 ± 0.1
PW, cm	0.9 ± 0.1
LVMi, g/m^2^	84.5 ± 15.8
EF, %	58.9 ± 2.8
GLS, %	−18.8 ± 1.6
GLD, mm	11.7 ± 1.5
E/A ratio	1.9 ± 0.5
E deceleration, ms	168.1 ± 46.9
E/e’ ratio	5 ± 0.8

**Table 2:** Displacement and strain at different left ventricular levels in Group 1. Displacement is highest in basal segments and lowest in apical segments, consistent with reversed basal to apical gradient. Strain is lowest in basal segments and highest in the apical segments.

LV Segments	Displacement, mm	*p*-Value	Strain, %	*p*-Value
Basal segments	17.6 ± 3.5	<0.000001	−16.3 ± 4.7	<0.000001
Mid segments	11.5 ± 2.9	<0.000001	−19 ± 2.5	<0.000001
Apical segments	4.22 ± 1.7	<0.000001	−21.1 ± 4.6	<0.000001

**Table 3:** Regional peak longitudinal displacement versus longitudinal strain in Group 1. Displacement is highest in the inferior and posterior wall and lowest in the antero-septal wall. Strain is highest in the inferior wall and otherwise is similar over the left ventricular walls.

Left Ventricle Wall	Antero-Septal	Septal	Inferior	Posterior	Lateral	Anterior
Displacement, mm	11.5 ± 3.4	14.6 ± 3.8	16.9 ± 4.3	16.5 ± 4.3	14.3 ± 4.3	13.8 ± 4.2
*p*-value	0.001	<0.000001	<0002	0.555	0.007	0.469
Strain, %	−18.3 ± 2.5	−16.1 ± 4.7	−19.3 ± 2.4	−17.6 ± 3.4	−17.3 ± 3.1	−17.8 ± 5.5
*p*-value	0.510	<0.002	<0.0001	0.002	0.678	0.557

**Table 4:** Demographic and echocardiography characteristics of patients with left ventricular dysfunction [Group 2]. BSA—body surface area, LAVi—left atrial volume index, LVEDD—left ventricular end diastolic diameter, LVESD—left ventricular end systolic diameter, IVS—interventricular septum, PW—posterior wall, LVMi—left ventricular mass index, EF—ejection fraction, WMSi—wall motion score index, GLS—global longitudinal strain, and GLD—global longitudinal displacement.

Age, years	69.1 ± 14.1
Gender, m	23 (77%)
Height, cm	169.9 ± 7.3
Weight, kg	76.9 ± 15.0
BSA, m^2^	1.9 ± 0.2
LAVi, mL/m^2^	37.7 ± 12.3
LVEDD, cm	4.7± 0.8
LVESD, cm	3.1 ± 0.8
IVS, cm	1.2 ± 0.3
PW, cm	1.0 ± 0.2
LVMi, g/m^2^	102 ± 31.4
EF, %	33.9 ± 3.2
WMSi	2 ± 0.3
GLS, %	8.2 ± 2.3
GLD, mm	4.4 ± 1.7
E/A	1.3 ± 0.9
E deceleration, ms	178.9 ± 62.5
E/E’	12.8 ± 5.5

**Table 5:** Comparison of segmental longitudinal displacement. AS—anterior septum, S—septum, I—inferior segments, P—posterior segments, L—lateral segments, A—anterior segments, Ap—apex, 1—basal segments, 2—mid-ventricular segments, and 3—apical segments.

Segment	Displacement (mm) Healthy Subjects	Displacement (mm) LV Dysfunction	Lower Limit of Normal (5th percentile), mm	Abnormal Displacement Rate in LV Dysfunction Patients (%)
AS1	14.1 ± 2.4	6.5 ± 3.1	9.3	83
S1	17.3 ± 2.9	7.8 ± 3.1	11.5	90
I1	20.2 ± 3.0	9.0 ± 3.1	14.2	96.7
P1	19.6 ± 3.2	8.7 ± 3.9	13.2	90
L1	17.4 ± 3.1	6.6 ± 3.8	11.2	96.7
A1	17.2 ± 2.7	6.0 ± 3.8	11.8	96.7
AS2	8.9 ± 2.0	3.7 ± 2.5	4.9	63.3
S2	11.8 ± 2.2	4.5 ± 2.7	7.4	86.7
I2	13.6 ± 2.5	5.2 ± 2.2	7.6	83
P2	13.3 ± 2.4	3.9 ± 4.2	8.5	90
L2	11.2 ± 2.8	2.2 ± 3.5	5.6	90
A2	10.3 ± 2.2	2.3 ± 2.7	5.9	90
S3	3.7 ± 1.5	1.6 ± 2.0	2.2	70
I3	5.2 ± 1.8	0.8 ± 1.7	3	80
L3	4.9 ± 1.6	0.2 ± 2.5	2.9	90
A3	3.2 ± 1.7	0.8 ± 1.4	2.5	83
Ap	4.2 ± 0.8	4.1 ± 1.4	2.6	93.3

**Table 6:** Comparison of segmental strain. AS—anterior septum, S—septum, I—inferior segments, P—posterior segments, L—lateral segments, A—anterior segments, Ap—apex, 1—basal segments, 2—mid-ventricular segments, and 3—apical segments.

Segment	Strain (%) Healthy Subjects	Strain (%) LV Dysfunction	Lower Limit of Normal (5th percentile), %	Abnormal Rate of Strain in LV Dysfunction Patients (%)	Strain < 10%	Strain < 12%	Strain < 14%
AS1	−16.8 ± 2.0	−9.2 ± 5.1	−12.9	80	56.7	76.7	86.7
S1	−14.7 ± 2.4	−10.5 ± 3.2	−9.9	33.3	33.3	66.7	80
I1	−18.3 ± 2.3	−13.2 ± 5.1	−13.7	53.3	23.3	36.7	53.3
P1	−17.1 ± 3.8	−14.4 ± 5.9	−9.5	13.3	13.3	36.7	40
L1	−16.6 ± 3.4	−11.4 ± 5.2	−9.8	30	30	40	60
A1	−17.4 ± 2.7	−10.5 ± 5.0	−12.0	50	36.7	50	60
AS2	−19.8 ± 2.0	−7.5 ± 4.5	−15.8	93.3	83.3	90	90
S2	−18.4 ± 2.5	−9.0 ± 4.6	−13.4	86.7	46.7	73.3	86.7
I2	−20.2 ± 2.2	−11.6 ± 5.0	−15.8	76.7	33.3	50	60
P2	−18.1 ± 2.8	−11.0 ± 5.1	−12.5	60	33.3	40	73.3
L2	−18.1 ± 2.6	−7.2 ± 4.5	−12.9	93.3	73.3	93.3	93.3
A2	−19.4 ± 2.1	−6.8 ± 4.4	−15.2	96.7	76.7	90	96.7
S3	−22.9 ± 2.9	−3.4 ± 4.9	−17.1	100	80	90	100
I3	−22.0 ± 2.9	−4.3 ± 5.2	−16.2	100	83.3	90	96.7
L3	−20.6 ± 3.0	−3.4 ± 4.8	−14.6	100	93.3	96.7	100
A3	−21.0 ± 2.8	−1.3 ± 4.2	−15.4	100	96.7	100	100
A	−21.3 ± 2.8	−3.0 ± 4.4	−15.7	100	90	96.7	100

**Table 7:** Comparison of manual GLS values. GLSm—GLS measured manually using the method suggested in this work; GLSm(r)—GLS measured manually according to the reference; and GLS—GLS measured using the EchoPAC software.

GLS	Strain (%) Healthy Subjects	Strain (%) LV Dysfunction	Lower Limit of Normal (5th Percentile), %	Abnormal Rate of GLS in LV Dysfunction Patients (%)	Strain < 16%
GLSm	−18.5 ± 1.8	−8.9 ± 2.1	−14.9	100	100
GLSm(r)	−18.3 ± 1.8	−8.9 ± 2.0	−14.7	100	100
GLS	−18.8 ± 1.6	−8.8 ± 2.0	−15.6	100	100

## Data Availability

The data supporting the findings of this study are available from the corresponding author upon reasonable request.
